# Adsorption Properties and Mechanism of Cd^2+^ in Water by Zr-containing Silica Residue Purification

**DOI:** 10.3389/fchem.2018.00556

**Published:** 2018-11-23

**Authors:** Wanting Chen, Han Zhang, Yu Liang, Hao Ding, Sijia Sun

**Affiliations:** ^1^Beijing Key Laboratory of Materials Utilization of Nonmetallic Minerals and Solid Wastes, National Laboratory of Mineral Materials, School of Materials Science and Technology, China University of Geosciences, Beijing, China; ^2^School of Materials Science and Technology, Shenyang University of Chemical Technology, Shenyang, China

**Keywords:** Zr-containing silica residue purification, adsorption, Cd^2+^, kinetic model, isothermal models

## Abstract

Zirconium (Zr)-containing silica residue purification (ZSR-P) discharged from industrial production of ZrOCl_2_ was used as an adsorbent, and CdCl_2_ solution was used as the simulated wastewater containing cadmium ions (Cd^2+^). The properties and mechanisms of ZSR-P absorbing Cd^2+^ were studied. The results showed that ZSR-P had a good effect on the adsorption and removal of Cd^2+^ in water. The adsorption time, initial concentration of Cd^2+^, and pH of the solution had a significant effect on the adsorption behavior, whilst the pH value had the greatest effect amongst them. Under optimal conditions, the amount of Cd^2+^ adsorbed by ZSR-P was 43.1 mg/g. The isothermal adsorption conformed to the Langmuir adsorption model, and the adsorption kinetics conformed to the secondary adsorption rate model. In ZSR-P-Cd, Cd^2+^ was uniformly distributed on the surface of SiO_2_ particles and in the pores formed by the accumulation of particles. Adsorption of Cd^2+^ by ZSR-P was achieved through the reaction between Si-OH on the surface of SiO_2_ and Cd^2+^ hydroxyl compounds.

## Introduction

In recent decades, especially in some developing countries, the world economy has been growing rapidly. The process of industrialization, agricultural economy, and urbanization have been speeding up, which results in heavy metal pollution in water due to mining, metal smelting process, electroplating, and pesticide and fertilizer abuse. The excess emissions of sewage have become more critical than ever (Sekhar et al., 2004; Srivastava and Majumder, 2008; Fu and Wang, 2011; Huang et al., 2014). Heavy metal ions are the main water pollutants. They are easy, biologically enriched and hard to degrade and have high toxicity and stable chemical property, so their damage is extremely serious (Sha et al., 2010). Cadmium ion (Cd^2+^) is one of the most hazardous ions among these heavy metal ions. It has a strong chemical activity in the environment and can easily migrate into the food chain and endanger human health (Solenkova et al., 2014; Venza et al., 2014). Chronic exposure to Cd could cause great damage to cardiovascular system, nervous system, and livers of mammals and humans and may lead to renal failure and ultimately death (Romero-Gonzalez et al., 2001). For this reason, international regulatory agencies have issued wastewater standards for the discharge of various heavy metal ions, including Cd, to limit their entry into surface water and municipal sewers. However, due to the high cost of compliance with pollution control legislation, some small businesses cannot afford it. Therefore, some efficient, convenient, and cost effective processes need to be found for removing heavy metal ions from wastewater.

Currently, there are many available methods to remove heavy metals from the environment (Basci et al., 2004; Dabrowski et al., 2004; Ho and Ofomaja, 2006; Janin et al., 2009; Soylak et al., 2010), including oxidation reduction, chemical precipitation, ion exchange, membrane separation and adsorption, etc. Amongst these methods, oxidation reduction and chemical precipitation are traditional methods which cause some issues such as the large amount of chemical reagents, a secondary source of pollution to the environment, and poor treatment of low-concentration wastewater. Although the methods of ion exchange and membrane separation are effective, the processing scale is small and both the investment cost and operation cost are high. In contrast, the adsorption method, which is highly efficient and simple, suitable for renewable use and is applicable for low-concentration wastewater, requires low investment. Therefore, it is an attractive method with a vital potential for further development. At present, in addition to some nonmetallic minerals, the adsorbents used to treat the wastewater containing heavy metal ions also include activated carbon, humic acid adsorbents, macromolecule materials, biomaterials, and industrial solid wastes (Wong et al., 2003; Sprynskyy et al., 2006; Fan et al., 2007; Veli and Alyüz, 2007; Mohammad-Khah and Ansari, 2009). Bhattacharyya and Gupta (2008), Zhu et al. (2009), and Hong-Ping et al. (2001) studied the adsorption behavior of Cd^2+^ by using montmorillonite and kaolinite with the adsorption method and found that the adsorption capacity of different minerals for Cd^2+^ was different. However, since the surface area of the mineral material is not high, the amount of adsorption is reduced; therefore, a large amount of the adsorbent is required for the operation, which may cause a certain proportion of resources to be wasted. Bayat (2002) used fly ash as an adsorbent to adsorb Zn^2+^ and Cd^2+^ in wastewater. Because of its porous honeycomb structure and large surface area in the internal structure, it has a good adsorption capacity for various metal ions in wastewater. The unmodified fly ash needs to be chemically modified in order to improve its adsorption performance as the small number of functional groups contained on the surface invisibly increases the processing cost of the adsorption process, thus complicating it. The adsorption capacity of the modified fly ash can be compared with that of activated carbon, and the cost is only one third of the activated carbon.

Recently, more attention has been paid to low-cost adsorbents that can be used to replace carbonaceous and mineral materials. Industrial solid waste has become a hot adsorbent material because of its dual advantages in cost and technology. Zirconium (Zr)-containing silica residue is the solid gelatinous waste during industrial acid reaction process in the production of zirconium oxychloride (ZrOCl_2_) from zirconite as the raw material. It is a loose-type gel. The main solid components of the gel are amorphous SiO_2_ and a small amount of unreacted zirconite, crystalline NaCl, and other impurities. The main liquid components are water, some dissolved substances such as hydrochloric acid (HCl), ZrOCl_2_, Na^+^, Cl^−^, and Fe^3+^, Zr^4+^, etc. In addition, the Zr-containing silica residue is purified to obtain Zr-containing silica residue purification (ZSR-P), which is mainly composed of amorphous SiO_2_. It is expected to be an excellent adsorbent for the treatment of heavy metal ions in the water by means of the surface charge properties of SiO_2_, the characteristics of functional groups, and the pore channels formed by the accumulation of particles. This work undoubtedly has a positive significance for recycling solid waste and reducing the cost of wastewater treatment. However, very few reports have been made on the adsorption behavior and the thermodynamics of heavy metal ions by ZSR-P.

In this work, the adsorption behavior of Cd^2+^ from an aqueous solution using ZSR-P was studied by a set of experiments at various conditions, including temperature, adsorption time, initial concentration of Cd^2+^, and initial pH value. In order to identify the possible mechanisms involved in Cd^2+^ binding by ZSR-P, some characterization methods such as Fourier transform infrared spectrometer (FTIR) and X-ray photoelectron spectroscopy (XPS) were performed along with the analysis.

## Experiments

### Raw materials, reagents, and instruments

**Figure 1:**
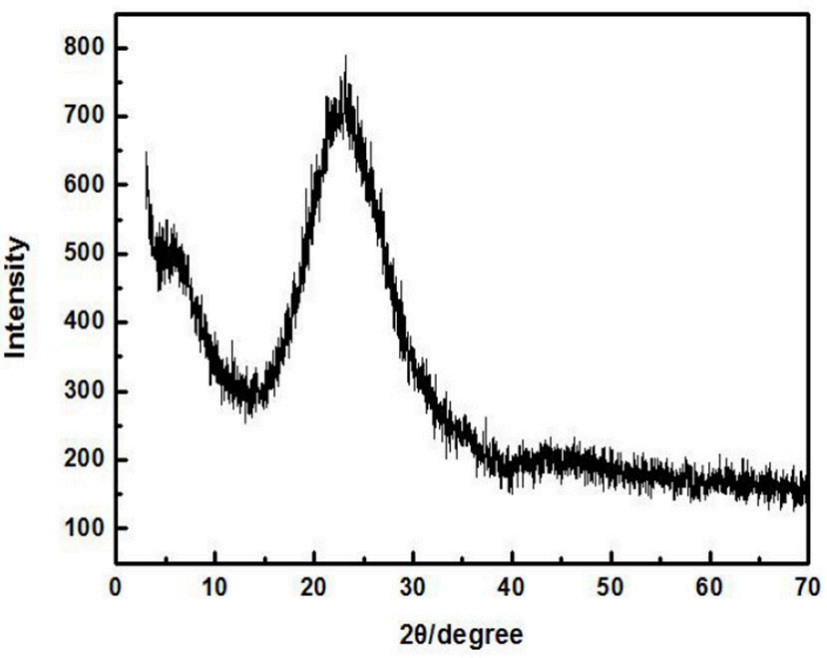
XRD of ZSR-P.

Zirconium-containing silica residue purification was obtained by purifying Zr-containing silica residue using a physical method. The Zr-containing silica residue raw material was produced by Henan Baililian Chemical Co. Ltd., Henan, China. The purification process involved the following steps. (1) The Zr-containing silica residue and water were added to a dispersing machine at a solid-liquid ratio of 7% to be crushed and mixed to form a uniform slurry. (2) The mixed slurry was pre-ground by an ultrafine grinder at 1,200 r/min. After 10 min, the alumina grinding balls were added to the mixed slurry at a ball ratio of 15:1. The grinding time was 35 min. (3) The mixed slurry containing the grinding balls was sieved to obtain the slurry. (4) The mixed slurry was separated by centrifugation, and each group of samples was centrifuged three times in order to obtain a gel of high purity containing amorphous silica. (5) The gel obtained after centrifugation was dried to finally obtain ZSR-P (Zhang et al., 2011). Additionally, ZSR-P had a specific surface area of 680.08 m^2^/g, and the pore diameter was 3.83 nm. The main chemical components before and after purification are shown in Table 1. The X-ray diffraction (XRD) analysis of ZSR-P is shown in Figure 1. It can be seen that a broad characteristic peak appears at a diffraction angle (2θ) of 20–25°, indicating that ZSR-P contains a large amount of amorphous phase components. In addition, there are no characteristic peaks of other impurities in the XRD pattern. So, combining with chemical analysis results (SiO_2_ content of 94.63%), it suggests that the main component of ZSR-P should be amorphous SiO_2_. Obviously, the properties of amorphous SiO_2_ determine the performance of ZSR-P.

**Table 1:** Comparison XRF of ZSR-P before and after purification.

**ZSR**		**ZSR-P**	
**Analyte**	**Result (%)**	**Analyte**	**Result (%)**
SiO_2_	81.8407	SiO_2_	92.7501
ZrO_2_	10.0438	ZrO_2_	4.7031
Cl	4.5786	Cl	1.2328
Na_2_O	1.2677	Al_2_O_3_	0.6939
CaO	0.4332	HfO_2_	0.2300
HfO_2_	0.3437	Na_2_O	0.0951
Fe_2_O_3_	0.3437	TiO_2_	0.0879
TiO_2_	0.3208	Fe_2_O_3_	0.0733
Al_2_O_3_	0.2688	Cr_2_O_3_	0.0545
SO_3_	0.2032	K_2_O	0.0411
MgO	0.1429	ThO_2_	0.0288
Y_2_O_3_	0.0809	NbO	0.0094
Cr_2_O_3_	0.0650	
ThO_2_	0.0396	
K_2_O	0.0272	

The CdCl_2_ solution was used as the simulated wastewater containing Cd^2+^. A certain amount of solid CdCl_2_ was weighed and added into distilled water, followed by stirring and dissolving to prepare the CdCl_2_ solution with different Cd^2+^ contents. An NaOH solution of 0.1 mol/L or HCl of 0.1 mol/L was used for pH adjustment. All chemicals used were of analytical reagent grade and were obtained from Beijing Chemical Works, China.

The main test equipments used are described as follows. The adsorption reaction of ZSR-P on heavy metal simulated wastewater under different conditions was carried out by using a SHZ-82 Thermostatic Bath Oscillator (Beijing Guohua Technology Group Ltd., Beijing, China). In addition, pHS-3C (Inesa Analytical Instrument Co., Ltd, Shanghai, China) was used to adjust the pH of the reaction solution. Prodigy Inductively Coupled Plasma Atomic Emission Spectrometry (Leeman Labs INC., USA) was used to test the residual heavy metal ion content after the adsorption reaction. The potential of the surface before and after the adsorption of heavy metals by the adsorbent was tested by using a Zetasizer Nano ZS Potentiometer (Malvern Panalytical, UK). The structural characteristics of mesoporous materials were characterized by a specific surface area and porosity analyzer (BET, Quadrasorb Si-MP, Quantachrome, USA). The characterization was mainly at liquid nitrogen temperature (77K), and the relative pressure was P/Po = 0~0.35. The particle size, surface morphology, and elemental content distribution of ZSR-P were determined by using a scanning electronic microscope combined with an energy dispersive X-ray detector (SEM-EDX, Hitachi High-Technologies Corporation, Tokyo, Japan). The particle size of the sample material and the comparative sample was observed using a FEI Tecnai G^2^F30 Field transmission electron microscope (TEM; acceleration voltage of 300 kV). The XRD analyses were conducted on a Rigaku D/max-rA (12 KW) X-ray powder diffractometer (XRD, D/MAX-2000, Rigaku Corporation,Tokyo, Japan) to determine the mineral composition operated with Cu Kαradiation at 40 kV and 100 mA and with a scanning speed of 0.5° (2θ)/min. A Fourier transform infrared spectroscope (FTIR, Perkin Elmer, Shanghai, China) was used to evaluate the distribution of functional groups on the surface between different materials. The XPS (Kratos Axis Ultra, UK) was used to characterize the surface composition and the combination state of the elements of ZSR-P before and after adsorption. Binding energies were referenced to the C1s peak at 284.8 eV. The oxide content and the elemental composition of the samples were analyzed by X-ray fluorescence (XRF) spectroscopy (S4-Explorer, Brukeraxs, Germany).

### Adsorption experiments

The 100 ml Cd^2+^ solution with a known concentration was weighed and transferred into a 250 mL Erlenmeyer flask, regulating the pH value and adding a certain amount of ZSR-P into the suspension. The suspension was shaken for a certain period of time and then centrifuged to obtain a precipitate and a supernatant liquid, which were the ZSR-P products adsorbing Cd^2+^ (ZSR-P-Cd) and the Cd^2+^ removed from the solution, respectively. The Cd^2+^ content in the supernatant can be measured to evaluate the adsorption effect.

### Characterization and evaluation method

1. Calculation of adsorption amount
  Inductively coupled plasma atomic emission spectrometry (ICP-AES) was used to determine the Cd^2+^ concentration of the supernatant after adsorption. The adsorption amount of Cd^2+^ by ZSR-P was calculated by the following formula:
  (1) $$\begin{array}{l} q=\frac{\left(C_{0}-C\right)V}{m}, \end{array}$$
  where C_0_ and C are the initial concentration and residual concentration of Cd^2+^ in the solution, respectively, which are also called the concentrations before and after adsorption by ZSR-P (mg/L); q is the adsorption capacity of Cd^2+^ adsorbed by ZSR-P (mg/g); V is the solution volume (L); and m is the quality of ZSR-P added (g).
2. Surface hydroxyl test
  An amount of 2 g of the sample to be tested was weighed and placed in a 200 mL beaker, adding 75 mL of NaCl solution, which had a quality score of 0.2; 25 mL of absolute ethanol was added to the mixture, and then the mixture was stirred evenly with a magnetic stirrer. After adjusting the pH of the solution to 4 by using 0.1 mol/L NaOH or 0.1 mol/L HCl, 0.1 mol/L NaOH was added to raise the pH of the solution to 9 slowly and the pH value was kept unchanged for 20 s. The volume of NaOH used was recorded, and the number of hydroxyl groups N per square nanometer of the sample was calculated according to the following formula:
  (2) $$\begin{array}{l} \text{N}=\frac{\text{CV}{\text{N}}_{\text{A}}}{\times }10^{-3}S\text{m}, \end{array}$$
  where C is the concentration of the NaOH solution (mol/L); V is the volume (L) of the NaOH solution; N_A_ is Avogadro's constant; S is the specific surface area of the sample (m^2^/g); and m is the mass of the sample (g).

## Results and discussion

### Study on adsorption properties

#### Effect of temperature

**Figure 2:**
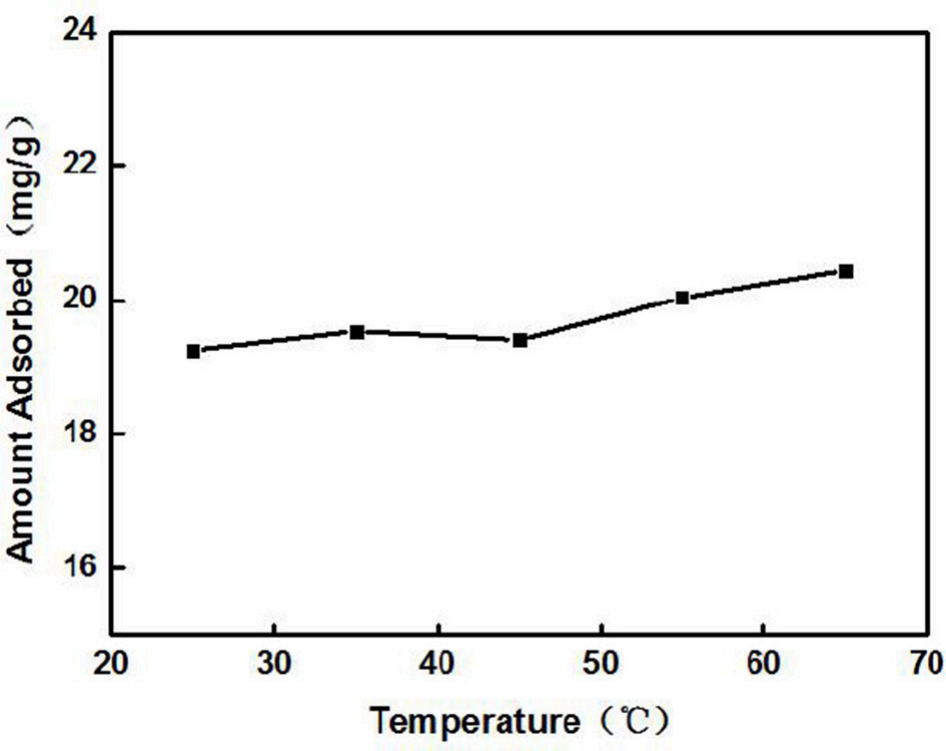
Effect of solution temperature on adsorption of Cd^2+^ by ZSR-P.

The Cd^2+^ solution with a concentration of 100 mg/L (actual measured value was 94.97 mg/L by ICP-AES) was adjusted to an initial pH of 8. Later, 3 g/L of ZSR-P was added and heated to a certain temperature; the thermal insulation oscillation time was 10 min. Figure 2 shows the effect of solution temperature on the adsorption of Cd^2+^ by ZSR-P. It can be seen that as the temperature rose from 25°C (room temperature) to 45°C, the amount of Cd^2+^ adsorbed by ZSR-P remained unchanged. When the temperature rose to 65°C, the adsorption amount increased slightly, indicating that temperature had little effect on the adsorption of Cd^2+^ by ZSR-P. According to the test results, considering the cost and operation conveniences for the treatment of the actual wastewater, the adsorption of the Cd^2+^ solution should be carried out at a temperature of 25°C, which is the normal temperature.

#### Adsorption kinetics

**Figure 3:**
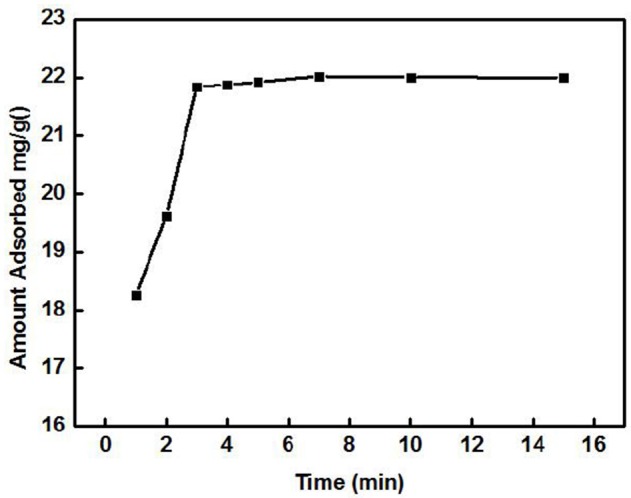
Effect of solution time on adsorption of Cd^2+^ by ZSR-P.

The effect of the adsorption time on the adsorption of Cd^2+^ by ZSR-P is shown in Figure 3. The concentration of the Cd^2+^ solution used was 100 mg/L (actual measured value was 106.87 mg/L by ICP-AES), initial pH was 8, temperature was 25°C, and ZSR-P dosage was 3 g/L. It can be seen from Figure 3 that ZSR-P showed a fast adsorption behavior when the adsorption time was between 0 and 3 min. The adsorption time exceeded 3 min, and the adsorption capacity increased slowly and reached an equilibrium value at 7 min; the value was 22 mg/g. It showed that ZSR-P had a good effect on Cd^2+^ adsorption, and the effect of Cd^2+^ removal from the water was obvious.

According to the adsorption data of Cd^2+^ by ZSR-P in Figure 3, fitting was performed according to Lagergren quasi-first-order kinetic model and quasi-second-order kinetic model. The equations of the two models are as follows:

(3) $$\begin{array}{l} \text{Quasi}-\text{first}-\text{order kinetic model}:\log\left(Qe-Qt\right)=\log Qe\\ -\frac{K_{1}}{2.303}•t \end{array}$$

(4) $$\begin{array}{l} \text{Quasi}-\text{second}-\text{order kinetic model}:\frac{t}{Qt}=\frac{1}{2K_{2}•{Q_{e}}^{2}}\\ +\frac{t}{Qe} \end{array}$$

In the formula, Qe is the quality (mg/g) of Cd^2+^ adsorbed by ZSR-P in solution during adsorption equilibrium; Qt is the quality (mg/g) of Cd^2+^ adsorbed by ZSR-P in solution at adsorption time t; and K1 and K2 are empirical factors.

**Figure 4:**
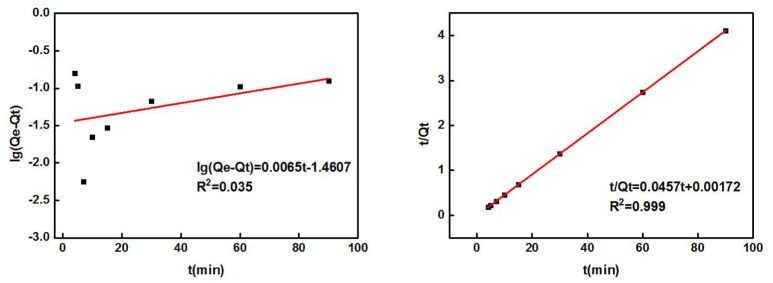
ZSR-P adsorption of Cd^2+^ kinetic equation fitting results. **(A)** Quasi-first-order kinetic model; **(B)** quasi-second-order kinetic model.

Table 2 shows the parameters of the fitted kinetic equations. Figure 4 compares the fitting curves and the distribution of the experimental data points. According to the quasi-first-order kinetic model, the correlation coefficient *R*^2^ was only 0.035, indicating a low level of fitting degree. According to the quasi-second-order kinetic model, *R*^2^ was as high as 0.999, indicating a high fitting degree. Obviously, the adsorption of Cd^2+^ by ZSR-P conformed to the pseudo-second-order kinetic model, and it also indicated that this adsorption was the surface adsorption reaction control process (Ho et al., 2000).

**Table 2:** ZSR-P adsorption kinetic parameters of Cd^2+^.

**Quasi-first-order kinetic model**			**Quasi-second-order kinetic model**		
**Qe (mg/g)**	***K*_1_**	***R*^2^**	**Qe (mg/g)**	***K*_2_**	***R*^2^**
28.89	0.015	0.035	21.88	0.607	0.999

#### Equilibrium isotherms

**Figure 5:**
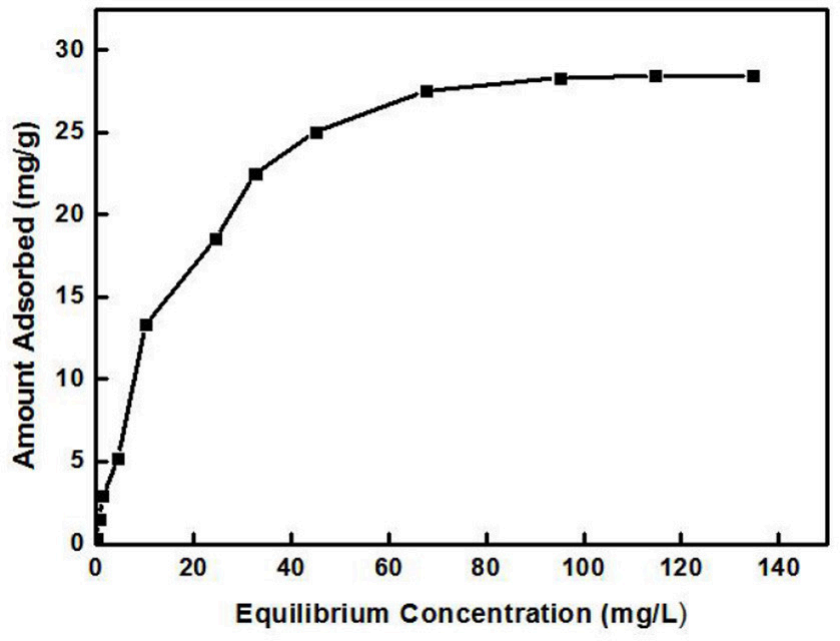
Adsorption isotherm equations of Cd^2+^.

Figure 5 shows the relationship between Cd^2+^ residual concentration (equilibrium concentration) in the solution and Cd^2+^ adsorption amount under the conditions of solution temperature 25°C, pH 8, ZSR-P dosage 3g/L, and adsorption time 15 min (In order to ensure that the adsorption reaction of Cd^2+^ by ZSR-P was fully balanced, the adsorption time was increased to 15 min), which is called the adsorption isotherm. It can be seen that with the increase in the concentration of Cd^2+^, the adsorption amount of Cd^2+^ by ZSR-P continued to increase until the concentration of Cd^2+^ was more than 80 mg/L; the stable value of the adsorption amount was 28 mg/g. According to the data in Figure 5, the isotherm of Cd^2+^ adsorbed by ZSR-P can be model-regressed. The Langmuir and Freundlich adsorption isothermal models commonly used are as follows.

Langmuir equation:

(5) $$\begin{array}{l} \frac{C_{e}}{Q_{e}}=\frac{1}{K_{L}Q_{m}}+\frac{C_{e}}{Q_{m}} \end{array}$$

Freundlich equation:

(6) $$\begin{array}{l} \ln\;Q_{e}=\ln\;K_{f}+\frac{1}{n}\ln\;C_{e} \end{array}$$

In the formula, Ce is the equilibrium concentration (mg/L) of Cd^2+^ adsorbed by ZSR-P in solution, Qe is the adsorption amount (mg/g), Qm is the saturated adsorption amount (mg/g), and K_L_, K_f_, and n are all empirical coefficients.

**Figure 6:**
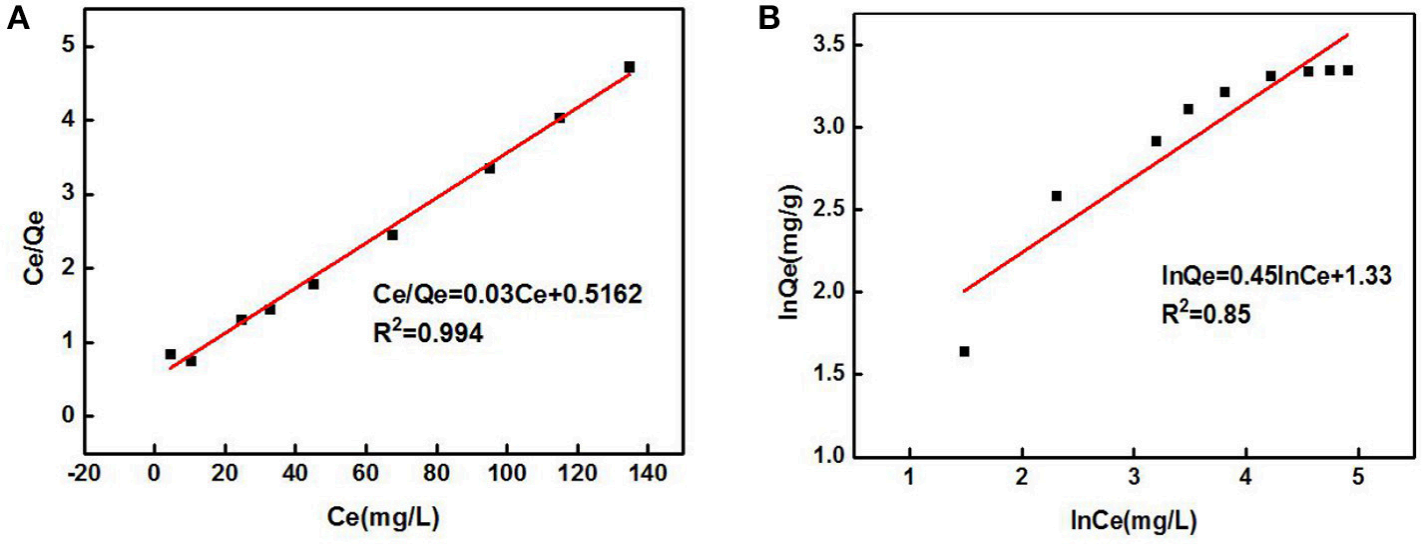
Adsorption isothermal models of Cd^2+^ adsorption by ZSR-P. **(A)** Langmuir model fitting results. **(B)** Freundlich model fitting results.

The data in Figure 5 were processed and model-fitted to obtain the adsorption isotherm equations described by the Langmuir and Freundlich models, as shown in Table 3 and Figure 6. It can be seen that the correlation constants *R*^2^ of the fitted Langmuir and Freundlich adsorption models were 0.994 and 0.85, respectively, indicating that the Langmuir model can better describe the adsorption isothermal behavior on Cd^2+^ by ZSR-P. The above results indicated that the adsorption of Cd^2+^ by ZSR-P was a monolayer adsorption, which indicated that the adsorption site was a single layer arrangement. The adsorption constant K_L_ was 0.058 (the value was small), indicating that the adsorption sites have a strong affinity (Langmuir, 2015).

**Table 3:** Results of the fitting models of Cd^2+^ adsorption by ZSR-P.

**Langmuir model**			**Freundlich model**		
***Q_*m*_***	***K_*L*_***	***R*^2^**	***n***	***K_*f*_***	***R*^2^**
33.33	0.058	0.994	2.22	3.78	0.85

#### Effect of pH

**Figure 7:**
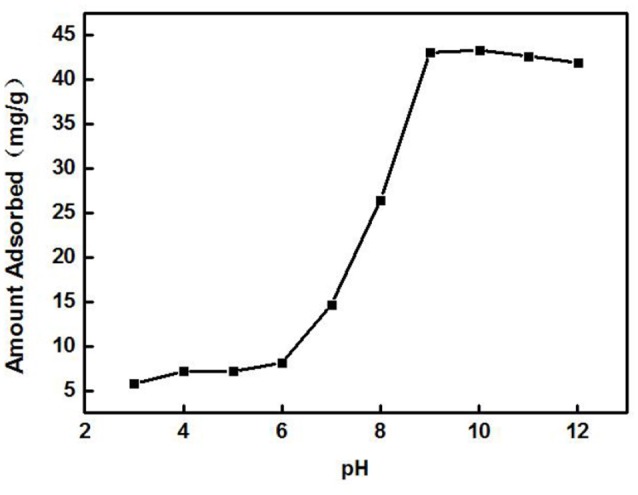
Effect of pH on the adsorption of Cd^2+^ by ZSR-P.

A 3 g/L solution of ZSR-P was added to the Cd^2+^ solution with different pH values under the conditions of solution Cd^2+^ concentration of 100 mg/L (actual measured value was 108.39 mg/L by ICP-AES) and temperature of 25°C, and then it was oscillated for 10 min to perform adsorption. Figure 7 shows the effect of solution pH on adsorption. From Figure 7, it can be seen that the solution pH had a great influence on the adsorption of Cd^2+^ by ZSR-P. When pH value was less than 6, the adsorption amount of Cd^2+^ was less than 10 mg/g, indicating that when pH was low, the adsorption of Cd^2+^ by ZSR-P was poor. When the pH was higher than 7, the adsorption amount increased greatly, and the maximum value was reached at pH 9, which was 43.1 mg/g. It showed that increasing the pH of the Cd^2+^ solution can greatly enhance the adsorption of Cd^2+^ by ZSR-P. The main reason was that Cd^2+^ was prone to hydrolysis at a higher pH, while ZSR-P has a stronger affinity to Cd^2+^ hydrolysate than free Cd^2+^ (Bayat, 2002). Obviously, the pH value of the solution was crucial for the adsorption of Cd^2+^ by ZSR-P.

## Adsorption mechanism

### Analysis of adsorption position

**Figure 8:**
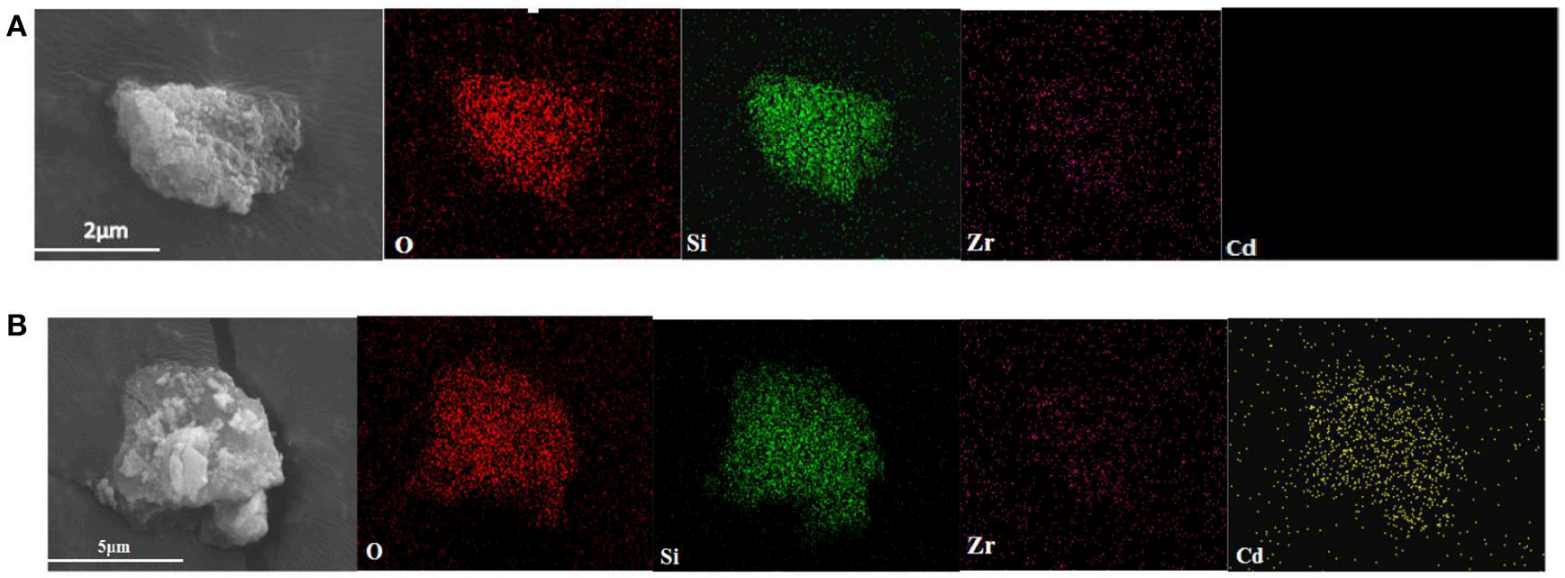
SEM images of ZSR-P **(A)** and adsorbed Cd^2+^ product **(B)**.

Figure 8 shows the SEM images and the surface distribution of the elements, such as O, Si, Zr, and Cd, of ZSR-P raw material and adsorbed Cd^2+^ product (ZSR-P-Cd). Table 4 shows the energy spectrum analysis (EDX) values of each element on the surface. It can be seen that there was no Cd in the ZSR-P raw material while there was a large amount of Cd in ZSR-P-Cd, which was consistent with the test results of the adsorption amount of Cd^2+^. The distribution area of Cd was consistent with the position of ZSR-P particles in the SEM images, indicating that Cd^2+^ was uniformly adsorbed on the surface of SiO_2_ particles in ZSR-P.

**Table 4:** Elemental analysis of ZSR-P and adsorbed Cd^2+^ product.

**Sample**	**O**		**Si**		**Zr**		**Cd**	
	**Atom fraction**	**Weight fraction**	**Atom fraction**	**Weight fraction**	**Atom fraction**	**Weight fraction**	**Atom fraction**	**Weight fraction**
ZSR-P	78.42	59.17	17.47	23.14	4.11	17.69	0	0
ZSR-P-Cd	76.90	54.64	18.10	22.58	2.32	9.41	2.68	13.37

**Figure 9:**
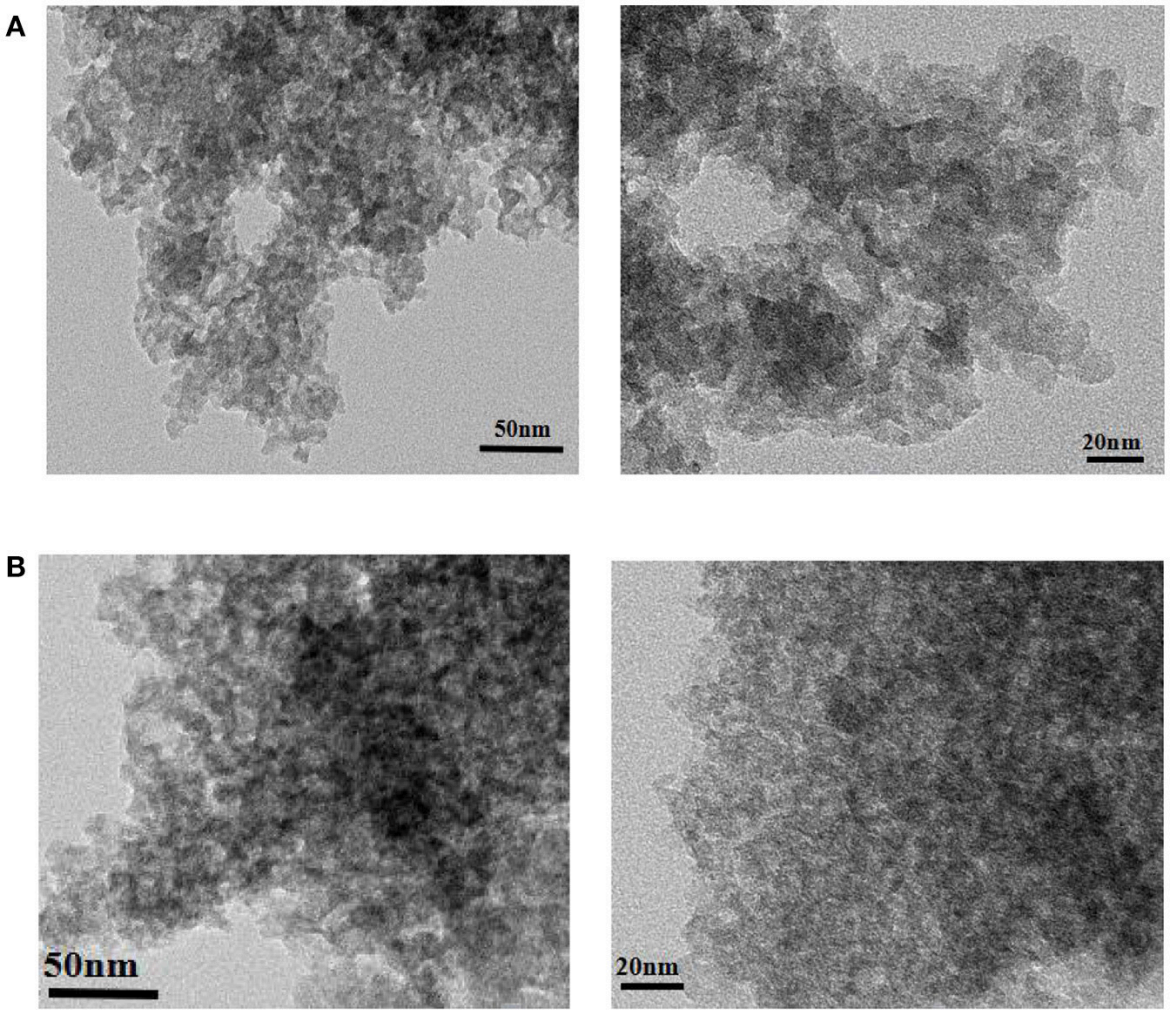
TEM images of ZSR-P **(A)** and adsorbed Cd^2+^ product **(B)**.

Figure 9 shows the TEM images of ZSR-P and ZSR-P-Cd. It can be clearly seen from Figure 9A that ZSR-P is mainly composed of aggregates formed by combining primary particles with sizes of about 20 nm. The small particles adhere to each other, and large aggregates of particles were formed. Due to the small particle size of the primary particles, ZSR-P had a large specific surface area, which was consistent with the test results. The large amount of hydroxyl groups present on the surface may provide a large number of adsorption and reaction binding sites for Cd^2+^. Figure 9B shows the TEM images of ZSR-P-Cd. It can be seen that the aggregated shape of ZSR-P-Cd particles was consistent with that of ZSR-P, indicating that no new substances were produced in the adsorption reaction. It was inferred that Cd^2+^ was adsorbed on the surface of ZSR-P and binding occurs between their interfaces.

### Adsorbent properties

#### Changes of zeta potential

**Figure 10:**
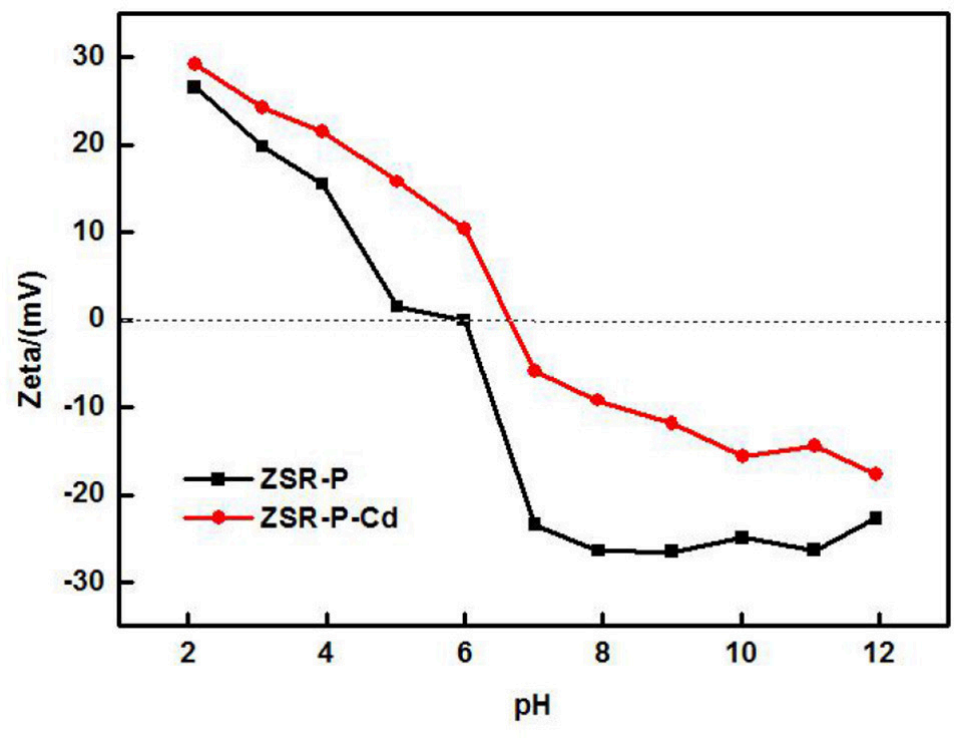
Zeta potential of ZSR-P and adsorbed Cd^2+^ product.

The zeta potential is the difference between the shear plane of the solid-liquid relative movement and the potential inside the solution. The magnitude and the positive and negative values are determined by the solid surface properties, the Stern layer of the solid-liquid interface, the properties of the medium, and the concentration and nature of the ions in the solvation layer, which can reflect the difference in surface properties of ZSR-P before and after the adsorption of Cd^2+^ (Moreno-Castilla et al., 2000, 2001). Figure 10 shows the surface zeta potential values of ZSR-P raw materials and adsorbed Cd^2+^ products (ZSR-P-Cd) at different pH conditions. It can be seen that the zeta potential of the surface before and after the adsorption of Cd^2+^ by ZSR-P in the water system decreased with the increase in the pH value. When pH was less than 5.92, the zeta potential of the ZSR-P surface was positive, which indicated that the surface charge of ZSR-P was positive, and the counter ion of the Stem layer in the solution was negative. As the pH increased, the H^+^ ions in the solution gradually decreased, and the OH^−^ ions in the Stern layer and the diffusion layer of the ZSR-P surface gradually increased. The zeta potential of the ZSR-P surface was negative, and the absolute value gradually increased. When ZSR-P adsorbed Cd^2+^, the point of zero charge of ZSR-P-Cd shifted to the right to pH 6.7, indicating that more cations were adsorbed on the surface of the particles. When the pH was less than 6.7, the surface charge value of ZSR-P-Cd was larger than that of ZSR-P under the same pH condition. When the pH was greater than 6.7, the absolute surface charge value of ZSR-P-Cd was smaller than that of ZSR-P at the same pH.

Using the surface hydroxyl test method in formula (2), the surface hydroxyl groups before and after adsorption of Cd^2+^ by ZSR-P were 1.39/nm^2^ and 1.06/nm^2^, respectively. Therefore, it can be seen from the changes in surface zeta potential and hydroxyl density that under the same pH condition, the number of hydroxyl groups on the surface of the product after adsorption of Cd^2+^ were much smaller than that on the surface of ZSR-P. This indicates that when Cd^2+^ was added to the solution, ZSR-P was surrounded by a large number of cations in the solution, and the large amount of hydroxyl groups on the surface became the main binding site of the adsorption reaction; therefore, Cd^2+^ ions occupy the surface of the ZSR-P particles and the pores formed by the accumulation of particles.

#### Fourier transform infrared (FTIR) spectroscopy

**Figure 11:**
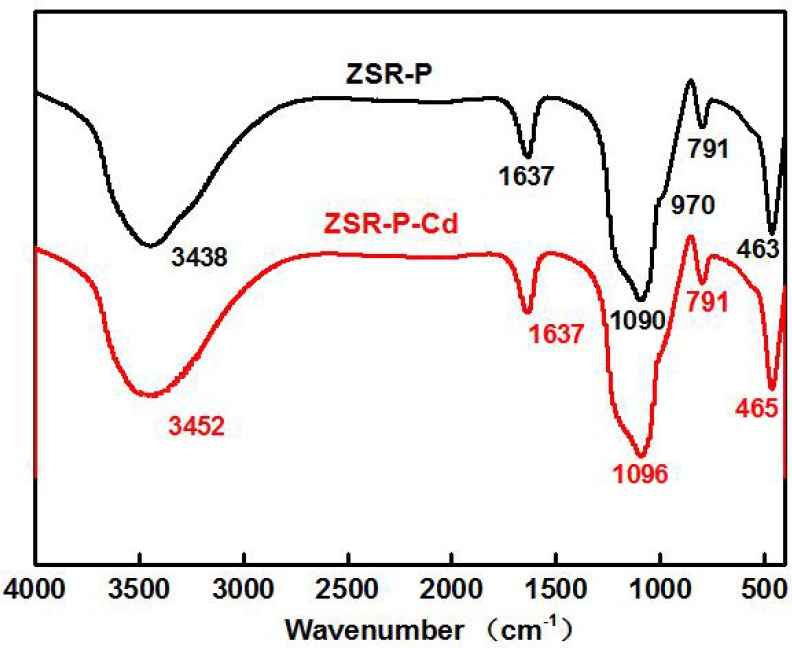
Infrared spectra of ZSR-P and ZSR-P-Cd.

Figure 11 is the infrared spectra of ZSR-P and its adsorbed Cd^2+^ product (ZSR-P-Cd). In the infrared spectrum of ZSR-P, the peak at 3,438 cm^−1^ was OH stretching of water; 1,637 cm^−1^ was OH deformation of water; the strong absorption band caused by Si-O stretching appeared at 1,090 cm^−1^ and 791 cm^−1^; the peak at 970 cm^−1^ belonged to Si-OH stretching; and the peak at 463 cm^−1^ was Si-O-Si deformation, reflecting the characteristics of amorphous SiO_2_ in ZSR-P. The peaks located at 1,637 cm and 3,438 cm were attributed by the abundant hydroxyl groups, which existing on the large specific surface area, absorb the surrounding water molecules. The comparison showed that the above absorption peaks appeared in the spectrum of ZSR-P adsorbed Cd^2+^ product except for the absorption peak at 970 cm^−1^, and the peaks at 1,090 cm^−1^ were slightly moved to 1,096 cm^−1^. It was speculated that the Si-O stretching (Da-Qing et al., 2000) formed on the surface of SiO_2_ hydrolyzed to form –SiOH (Bogdan et al., 1996) and combined with Cd^2+^ (d'espinose de la Caillerie et al., 1997). The disappearance of the Si-OH stretching at 970 cm^−1^ was presumed to be due to the dehydroxylation reaction on the surface of ZSR-P after the adsorption of Cd^2+^.

#### XPS analysis

**Figure 12:**
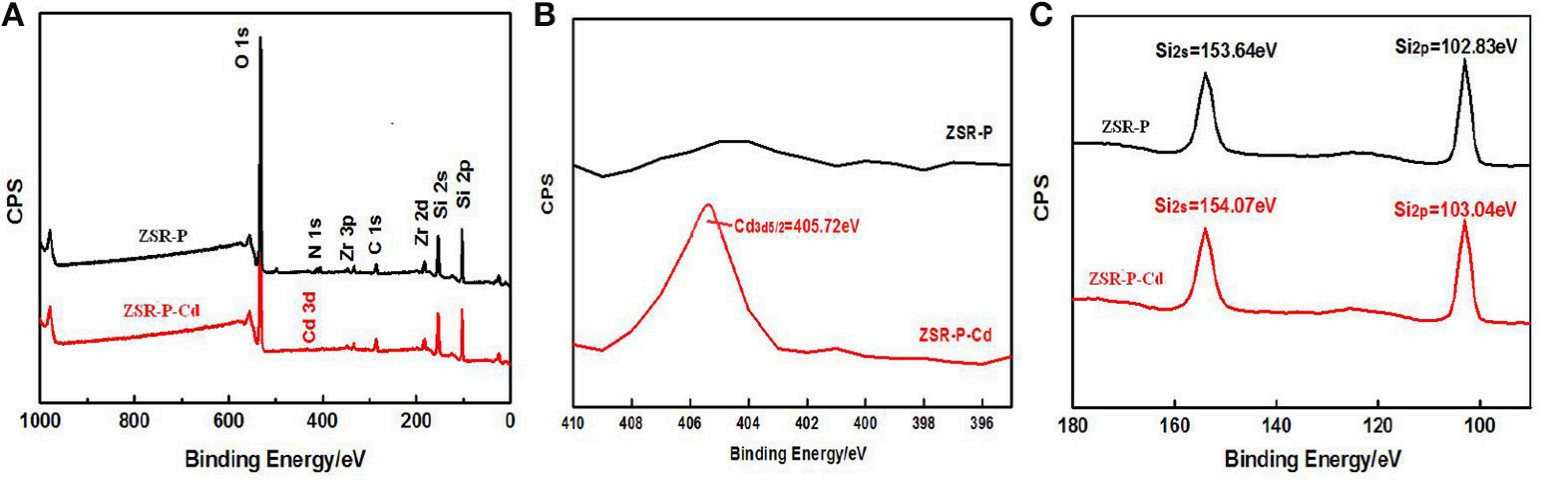
XPS of ZSR-P and ZSR-P-Cd. **(A)** The XPS full spectrum of ZSR-P and ZSR-P-Cd. **(B)** The Cd3d spectrum of ZSR-P-Cd. **(C)** The Si2s and Si2p spectra of ZSR-P and ZSR-P-Cd.

X-ray photoelectron spectroscopy analysis of ZSR-P and ZSR-P-Cd was performed, and the results are shown in Figure 12. The results show that the peaks of O, Si, and Zr appeared in the ZSR-P spectrum, reflecting the characteristics of ZSR-P being a component of SiO_2_ and a small quantity of Zr-containing impurities. However, the peak of Cd3d5/2 appeared in the XPS of ZSR-P-Cd, and its binding energy was 405.72eV, indicating that Cd^2+^ was adsorbed on the surface of SiO_2_, which was consistent with the results of SEM. In addition, the binding energies of Si2s and Si2p in the XPS of ZSR-P were 153.64 and 102.83 eV, respectively. While their binding energies in the XPS of ZSR-P-Cd were 154.07 and 103.04 eV, respectively. The larger binding energy value indicated that the chemical environment of Si had changed after the adsorption of Cd^2+^, which was consistent with the results of Si-OH on the SiO_2_ surface and Cd^2+^ hydrate reactions.

### Adsorption model

**Figure 13:**
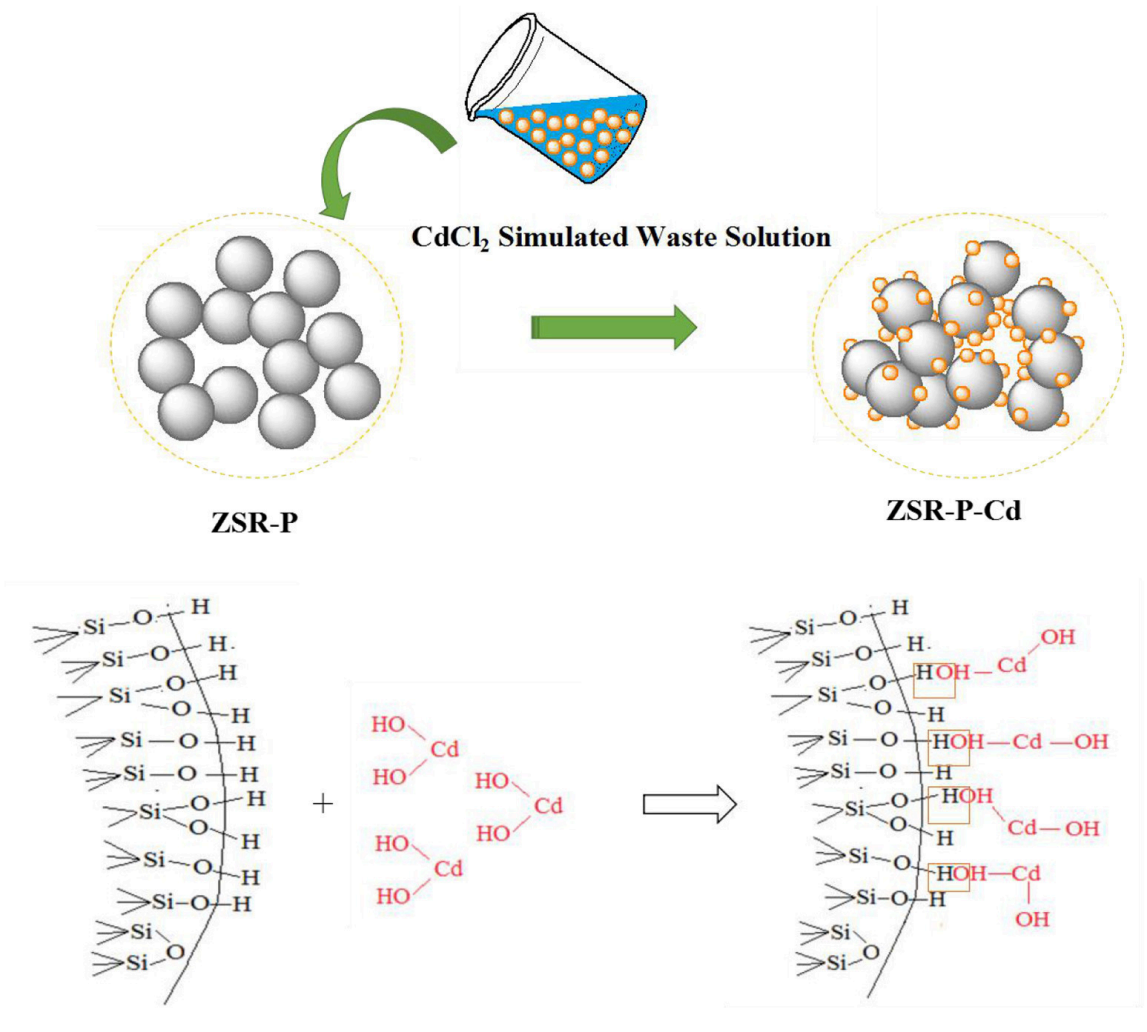
ZSR-P adsorption model of Cd^2+^.

Based on the above analysis of the properties and mechanisms of ZSR-P adsorbing Cd^2+^, an interaction model reflecting this property, mechanism, and process was established and shown in Figure 13.

## Conclusions

1. Zirconium-containing silica residue purification is mainly composed of aggregates of amorphous SiO_2_ nanoparticles. It has an excellent adsorption and removal effect on Cd^2+^ in water. The pH value of the solution, Cd^2+^ concentration, and adsorption time have a significant effect on the removal effect. In addition, ZSR-P, at a dosage of 3 mg/L, was added to an aqueous solution, which had a Cd^2+^ concentration of 100 mg/L and pH 9, and then the mixture was shaken for 10 min; subsequently, the adsorption reached an equilibrium. The adsorption amount of Cd^2+^ by ZSR-P is 43.1 mg/g.
2. The isothermal adsorption of Cd^2+^ by ZSR-P is in accordance with the Langmuir adsorption model, consistent with the adsorption characteristics of general microporous materials. The adsorption kinetics equation of ZSR-P for Cd^2+^ conforms to the second-order model for adsorption rate.
3. The removal of Cd^2+^ in aqueous solution by ZSR-P is mainly based on the adsorption between them. In addition, Cd^2+^ is uniformly distributed on the surface of SiO_2_ particles and in the pores formed by the accumulation of particles in ZSR-P-Cd. Adsorption between SiO_2_ and Cd^2+^ is achieved by the reaction between Si-OH on the surface of SiO_2_ and Cd^2+^ hydroxyl compounds.

## Author contributions

WC, HZ, HD, and SS conceived and designed the experiments. WC, HZ, and YL performed the experiments. WC and SS analyzed the data. HZ, YL, and SS contributed reagents. WC wrote the manuscript. YL retouched the document.

### Conflict of interest statement

The authors declare that the research was conducted in the absence of any commercial or financial relationships that could be construed as a potential conflict of interest.
